# Selective Grazing by a Tropical Copepod (*Notodiaptomus iheringi*) Facilitates *Microcystis* Dominance

**DOI:** 10.3389/fmicb.2018.00301

**Published:** 2018-02-23

**Authors:** Ewaldo Leitão, Kemal A. Ger, Renata Panosso

**Affiliations:** ^1^Graduate Program of Ecology, Federal University of Rio Grande do Norte, Natal, Brazil; ^2^Department of Microbiology, Federal University of Rio Grande do Norte, Natal, Brazil

**Keywords:** tropical zooplankton, eutrophication, cyanobacteria, facilitation, positive interactions, copepod, *Notodiaptomus*

## Abstract

Top-down grazer control of cyanobacteria is a controversial topic due to conflicting reports of success and failure as well as a bias toward studies in temperate climates with large generalist grazers like *Daphnia*. In the tropical lowland lakes of Brazil, calanoid copepods of the *Notodiaptomus* complex dominate zooplankton and co-exist in high abundance with permanent blooms of toxic cyanobacteria, raising questions for grazer effects on bloom dynamics (i.e., top-down control vs. facilitation of cyanobacterial dominance). Accordingly, the effect of copepod grazing on the relative abundance of *Microcystis* co-cultured with a eukaryotic phytoplankton (*Cryptomonas*) was evaluated in a series of 6-day laboratory experiments. Grazer effects were tested in incubations where the growth of each phytoplankton in the presence or absence of the copepod *Notodiaptomus iheringi* was monitored in 1 L co-cultures, starting with a 6-fold initial dominance of *Cryptomonas* by biomass. Compared to the no grazer controls, *N. iheringi* reduced the growth of both phytoplankton, but *Cryptomonas* growth was reduced to negative values while *Microcystis* growth continued positively despite grazers. Hence, in a matter of 6 days selective grazing by *N. iheringi* increased the biomass of *Microcystis* relative to *Cryptomonas* by an order of magnitude compared to controls, and thus, facilitated the dominance of this cyanobacterium. To account for the potential effect of allelopathy, we performed a secondary experiment comparing the abundance and growth rate of *Microcystis* and *Cryptomonas* in single and mixed co-cultures in the absence of grazers. The growth rate of *Microcystis* was unaffected by the presence or relative abundance of *Cryptomonas*, and vice versa, indicating no allelopathic effects. Our results suggest that selectively grazing zooplankton may facilitate cyanobacteria blooms by grazing on their eukaryotic phytoplankton competitors in nature. Given that selective grazers predominate zooplankton biomass in warmer waters, grazer facilitation of blooms may be a common but poorly understood regulator of plankton dynamics in a warmer and more eutrophic world.

## Introduction

Increasing frequency, duration, and range of cyanobacteria blooms reduce water quality and disrupt the flow of energy from primary production to higher trophic levels due to their poor nutritional quality, toxicity, and morphological defenses for zooplankton grazers (Dickman et al., 2008; Rastogi et al., 2015; Heathcore et al., 2016). While elevated nutrient concentrations and warm temperatures are recognized as key drivers of blooms (Smith and Schindler, 2009; Paerl and Otten, 2013), the trophic interactions in eutrophic systems, and especially the question of “who grazes the bloom,” is less understood (Urrutia-Cordero et al., 2015; Ger et al., 2016c). This is partly because of the bias toward studies in cooler temperate climates with relatively short bloom durations, where large and tolerant generalist grazers like *Daphnia* make top down control a possibility even under ideal abiotic conditions for cyanobacterial growth (Sarnelle et al., 2010; Chislock et al., 2013). Yet, in warmer climates with longer duration blooms, large generalist grazers are replaced with smaller and more selectively grazing zooplankton (Fernando, 1994; Hansson et al., 2007). Warmer, more eutrophic conditions are proliferating and predicted to expand globally (Paerl and Huisman, 2009; O'Neil et al., 2012). Consequently, trophic interactions in warmer and bloom-dominated waters deserve more attention, not only to better understand the ecology of cyanobacteria in tropical regions but also because they can be used as models for predicting future changes in temperate lakes (Ger et al., 2014; Paerl, 2017).

While cyanobacterial traits like toxicity and colonial morphology act as grazer defenses, grazing pressure on cyanobacteria also depends on the functional traits of zooplankton co-existing with blooms. Size, grazing behavior, and physiological tolerance to ingested cyanobacterial toxins are key traits controlling the degree of grazing pressure on blooms (DeMott et al., 1991; Kirk and Gilbert, 1992; Litchman et al., 2013). While generalist and tolerant grazers may reduce cyanobacterial blooms (Chislock et al., 2013), selective and tolerant grazers are expected to facilitate cyanobacteria by grazing on their eukaryotic phototrophic competitors (Scotti et al., 2015). Indeed, zooplankton that tolerates a limited ingestion of cyanobacteria while selectively grazing on their eukaryotic phytoplankton competitors has long been hypothesized to promote blooms (Kirk and Gilbert, 1992; Mitra and Flynn, 2006; Hong et al., 2013). This prediction, however, is largely based on short-term (<4 h) grazing experiments or modeling studies and remains untested experimentally at time scales above a few hours. Longer incubation periods would improve our understanding of the effects of selective grazers on cyanobacterial dominance. Specifically, longer incubations can be designed to account for phenotypic plasticity in grazing pressure (Ger et al., 2011) or cyanobacterial defenses (Jang et al., 2007); as well as effects due to starvation (e.g., prey switching) or mortality (e.g., cyanobacterial toxicity); and phytoplankton nutrient limitation or regeneration by zooplankton excretion.

High zooplankton biomass, especially calanoid copepods >50 ind.L^−1^, often co-exists with permanent blooms across tropical and subtropical lowland eutrophic lakes and reservoirs (Bouvy et al., 2001; Rietzler et al., 2002; Sousa et al., 2008). In tropical South America, calanoid copepods from the genus *Notodiaptomus* often dominate zooplankton communities, and their abundance can be positively correlated to toxic cyanobacteria blooms (Eskinazi-Sant'Anna et al., 2013; Rangel et al., 2016). This makes *Notodiaptomus* species ideal candidates to study plankton interactions in eutrophic waters characterized by long-duration cyanobacteria blooms. Moreover, recent experiments showed that ingestion of microcystin producing *Microcystis* had no effect on survival or egg production of the copepod *Notodiaptomus iheringi* in the presence of alternative nutritious prey, and identified both physiological tolerance and selective avoidance of *Microcystis* as key traits for co-existing with toxic cyanobacteria during blooms (Ger and Panosso, 2014; Ger et al., 2016b). Hence, the copepod *N. iheringi* exhibits zooplankton traits that can be expected to facilitate cyanobacteria.

To experimentally evaluate the facilitation of cyanobacteria by grazers across periods longer than a few hours, however, it is necessary to account for all factors controlling the competition between cyanobacteria and eukaryotic phytoplankton. Thus, in addition to herbivory (i.e., selective removal of a competitor), potential effects due to interference competition (e.g., allelopathy) must also be accounted for. Allelopathy is the release of chemical cues into the environment by an organism that inhibits the growth of a competitor (Rice, 1984; Leflaive and Ten-Hage, 2007; Dias et al., 2017).

Cyanobacteria can have allelopathic effects on other groups of phytoplankton including cryptophytes (B-Béres et al., 2012), chlorophytes (Bar-Yosef et al., 2010; Leão et al., 2010; Bittercourt-Oliveira et al., 2015), and other cyanobacteria (Mello et al., 2012; Zhai et al., 2013). Yet, eukaryotic phytoplankton can also release allelopathic compounds, and an established community of chlorophytes might also suppress the growth of cyanobacteria (Bittercourt-Oliveira et al., 2015). As a result, understanding eutrophic plankton communities and the trophic interactions controlling cyanobacterial dominance requires experiments that account for multiple biotic interactions such as grazing and allelopathy (Granéli et al., 2008).

Accordingly, we tested the hypothesis that selective grazing would facilitate the dominance of cyanobacteria by direct elimination of their eukaryotic phytoplankton competitors. For this, we evaluated the trophic interactions between the tropical copepod *N. iheringi* and two competing phytoplankton prey: the prokaryotic cyanobacteria *Microcystis* and the eukaryotic *Cryptomonas*. The effect of this grazer on *Microcystis* dominance was evaluated in a 6-day incubation experiment, where we compared the abundance and growth of co-cultured phytoplankton with and without copepods with a 6:1 initial dominance of *Cryptomonas* biomass. Although the main objective was to test grazer facilitation of cyanobacteria, we also performed a secondary experiment in order to rule out any allelopathic effects in the absence of grazers. This experiment tested if the presence and initial proportion of a competitor inhibited growth of either phytoplankton relative to single species cultures by comparing growth rates of each phytoplankton (*Microcystis* and *Cryptomonas*) in single and co-cultures across various initial biomass proportions.

## Methods

### Phytoplankton cultures

Cultures of *Cryptomonas obovata* (CCMA-UFSCar 148, WDCM 835) were initially obtained from the Federal University of São Carlos. The *Microcystis aeruginosa* (LEA-04) strain was obtained from culture collection at UFJF (Federal University of Juíz de Fora, Brazil), isolated in 2005 from Itumbiara reservoir, Goiás and is a microcystin producer strain. Under the culture conditions (below), this strain had a total microcystin quota of 30 fg MC.cell^−1^ (± 4 fg MC.cell^−1^, 95% confidence interval, hereafter indicated by ±, *n* = 3) based on an ADDA-ELISA assay (Enzo Science, USA) following the manufacturer protocol. Phytoplankton cultures were maintained as semi-continuous batch cultures grown in Wright's Cryptophyte (WC) medium in glass flasks at 23°C (± 1°C) under a 50-μmol quanta m^−2^ s^−1^ 12:12 h dark:light cycle. Cultures were kept at the exponential growth phase by weekly dilution into fresh media and only cells in this phase were used in experiments. Flasks were gently swirled regularly to prevent clusters of cells.

*Cryptomonas* grew as motile spheroid single cells (mean 12.5 × 6.5 μm) and *Microcystis* grew in spherical single or double cells (roughly 50% each) with a mean diameter of 4.5 μm. Carbon biomass content of cultures was estimated by multiplying cell density (via hemocytometer counts) and biovolume using the formulae: pgC cell^−1^ = 0.1204 × (μm ^3^)^1.051^ (Rocha and Duncan, 1985) as previously detailed (Ger et al., 2016b).

### Copepod culture

Individuals of *N. iheringi* were collected with a 64 μm mesh size surface tow in October 2015 from the mesotrophic Extremoz lagoon (5°42′25.8″S 35°16′56.2″W, Rio Grande do Norte, Brazil), with no history of cyanobacterial blooms at the time of sampling. Within 2 h of collection, live zooplankton were brought to the laboratory, concentrated on a 200 μm mesh, and transferred to a petri dish. Healthy (i.e., active and parasite free) gravid females of *N. iheringi* were isolated in drops and rinsed with distilled water, transferred to 2 L glass beakers filled with modified WC medium (lacking NO_3_ and PO_4_), to acclimate copepods for experimental conditions, and maintained at 23°C (± 1°C) with 500 μgC.L^−1^.d^−1^ of *Cryptomonas*, which was previously shown to be the optimum quantity to sustain cultures under these conditions. A new brood of copepods were grown and acclimated to these conditions prior to subsequent experiments. Only young adults and C5 copepodites from this brood were used in the experiments, which minimized potential age-related differences in the results.

### Alellopathy experiment

Allelopathy was quantified by comparing the growth rate of *Cryptomonas* and *Microcystis* in single species (i.e., control) versus mixed co-cultures (treatment) over a period of 6 days under identical conditions as the phytoplankton stock cultures explained above. Controls and treatments were prepared by diluting respective stock cultures into 150 mL WC medium at a total concentration equivalent to 0.35 mg C.L^−1^ (± 0.10 mg C.L^−1^, *n* = 15, Appendix Table 1A) in a 250 mL glass flask. There were three replicates each for the *Cryptomonas* (C) and *Microcystis* (M) controls, and nine treatments (C+M co-cultures) with a gradient in the initial carbon biomass ratio covering a range in the dominance of one phytoplankton to the other (C:M = 0.21, 0.22, 0.64, 1.12, 1.37, 1.81, 2.4, 2.67, 5.55). Experimental flasks were gently swirled three times a day to avoid cell clusters. Aliquots of 10–20 mL from each flask were sampled every two days (days 0, 2, 4, and 6) using sterile pipette tips in order to assess phytoplankton cell density and growth rate. Samples were preserved with 1% gluteraldehyde (final concentration), filtered on a 0.6 μm pore black polycarbonate filter (GE Water & Process Technologies), and counted using epi-fluorescent microscopy (Olympus/EX41) as described in Ger et al. (2016b). At least 100 *Cryptomonas* cells and 400 *Microcystis* cells were counted in either field or transects (Lund et al., 1958). The growth rate (R) of each phytoplankton species in the single and co-cultures during the experiment was measured using the difference in cell concentrations between the initial and final day (i.e., day 0 and 6), according to the formula R = ln(C_f_/C_i_)/days, where C_f_ and C_i_ is the final and initial cell concentration, respectively.

### Facilitation experiment

The effect of *N. iheringi* on the competition between each phytoplankton was quantified by comparing the cell density, growth rate, and relative abundance of *Cryptomonas* and *Microcystis* co-cultured with and without grazers over a period of 6 days. The no grazer control (–Z) and treatment with zooplankton (+Z) were identical except for the presence of *N. iheringi*, each were replicated four times, took place in glass flasks filled with 1 L of WC medium, and kept at 23°C (± 1°C) under a 50-μmol quanta m^−2^ s^−1^ 12:12 h dark:light cycle. Flasks were placed randomly on the incubator shelf in relation to the light source, and their position was randomly changed daily to minimize variability in phytoplankton growth due to potential differences in light availability. Flasks were prepared by diluting each phytoplankton type from exponentially growing stock cultures into freshly prepared sterilized medium with an initial mean *Microcystis:Cryptomonas* (M:C) carbon biomass ratio of 0.18 (± 0.05, n = 8) and a total concentration equivalent to 0.37 mg C.L^−1^ (± 0.09 mg C.L^−1^, *n* = 8, Appendix Table 1B), which was previously shown to be within the range of optimal food concentration for *N. iheringi* grazing (Ger et al., 2016b). Each treatment additionally received 50 adult *N. iheringi*, which were previously isolated in single droplets of distilled water and transferred into the flask by a wide mouthed plastic pipette. The same extra volume of distilled water added by this process was added also to the control flasks (~5 mL). Each flask was sealed with a 0.2 μm Millex FG vent filter (Millipore, USA), gently aerated (5 bubbles per second), and swirled gently three times a day to prevent cell clusters. Aliquots of 20 mL from each flask were collected every 2 days (days 0, 2, 4, and 6) using sterile equipment in order to quantify phytoplankton cell density (described above), from which growth rate (described above), relative abundance (described below), and clearance rates (described below) were calculated. The density of 50 copepod.L^−1^ corresponds to values observed in nature (Cabral, 2015). Copepods suspected of being dead were checked for mortality (i.e., motility and heart beat under a dissecting microscope) daily during experiment and dead ones were replaced.

The relative abundance of competing phytoplankton was calculated as the carbon equivalent biomass ratio of M:C estimated from the biovolume of each prey type as detailed above. The effect of grazers on the relative abundance of *Microcystis* was further evaluated by comparing the change in the M:C biomass ratio among treatments and controls (i.e., M:C_final_/M:C_initial_; where final and initial refer to the M:C at the end and start of the experiment, respectively).

To quantify grazing on each phytoplankton, prey-specific rates of clearance (CR) were calculated based on relative change in the initial and final cell concentration among treatments and controls through time (Frost, 1972), according to the following formula (Ger et al., 2011):

$$\begin{array}{l} \text{CR}=\left(\text{b}-\text{a}\right)\times \text{V}/\text{N}\\ \text{b}=\text{ln}\left({\text{C}}_{\text{f}}\;/\;{\text{C}}_{0}\right)\;/\text{ t}\\ \text{a}=\text{ln}\left({\text{T}}_{\text{f}}\;/\;{\text{T}}_{0}\right)\;/\text{ t} \end{array}$$

Where V is the culture volume (mL), N is the number of copepods, C_f_ is the final algal concentration (μg C.L^−1^) in controls, C_0_ is the initial algal concentration of the controls, T_f_ is the final algal concentration in the treatments, T_0_ is the initial algal concentration in the treatments, and t is the grazing period (in hours). Prey specific rates of ingestion (IR) were then calculated by multiplying prey specific CR with the average cell concentration (C) according to Frost (1972) using the following formula:

$$\begin{array}{l} \text{C}=\left({\text{T}}_{\text{f}}-{\text{T}}_{0}\right)\;/\text{ ln}\left({\text{T}}_{\text{f}}\;/\;{\text{T}}_{0}\right) \end{array}$$

We calculated the CR and IR for three different time periods (i.e., day 0–2; 0–4; 0–6) in order to compare potential differences during the experiment due to variable food concentration through time. Prey specific CR and IR are abbreviated as CR_C_ and IR_C_ for *Cryptomonas* and CR_M_ and IR_M_ for *Microcystis*. The effect of *N. iheringi* grazing on the carbon equivalent biomass of phytoplankton prey was further evaluated by comparing species specific and total phytoplankton biomass between controls and treatments at the start and end of the experiment. In all experiments, the effect of nutrient and light limitation on phytoplankton growth was assumed to be negligible due to the nutrient rich WC medium and exponential growth in the absence of grazers during the incubation period (see Results).

### Data analyses

Difference in phytoplankton concentration over time between controls and treatments were evaluated by comparing temporal trends in cell density and by comparing growth rates or change in biomass between controls and treatments (i.e., “single vs. co-culture” or “no-grazer vs. grazer” in the allelopathy and facilitation experiment, respectively). Quantile-quantile and residual plots were checked for normality and homoscedasticity. Normality of error and homogeneity of variance were also checked with a Shapiro Wilk and Barlett test, respectively. Non-normally distributed responses were log transformed. Linear regression was used to evaluate the effect of initial phytoplankton biomass ratio on the growth rate of each co-cultured phytoplankton in the allelopathy experiment. Differences in responses among controls and treatments (i.e., phytoplankton growth rate, final phytoplankton biomass, change in M:C biomass ratio) were evaluated by a Welch's *t*-test or Kruskal-Wallis test in case variances were not homogeneous after transformation. Difference in CR and IR estimates between the three time periods for each phytoplankton was compared with a Welch's *t*-test and one-way ANOVAs. All statistical tests were performed using R software (R Core Team, 2016).

## Results

### Allelopathy

**Figure 1:**
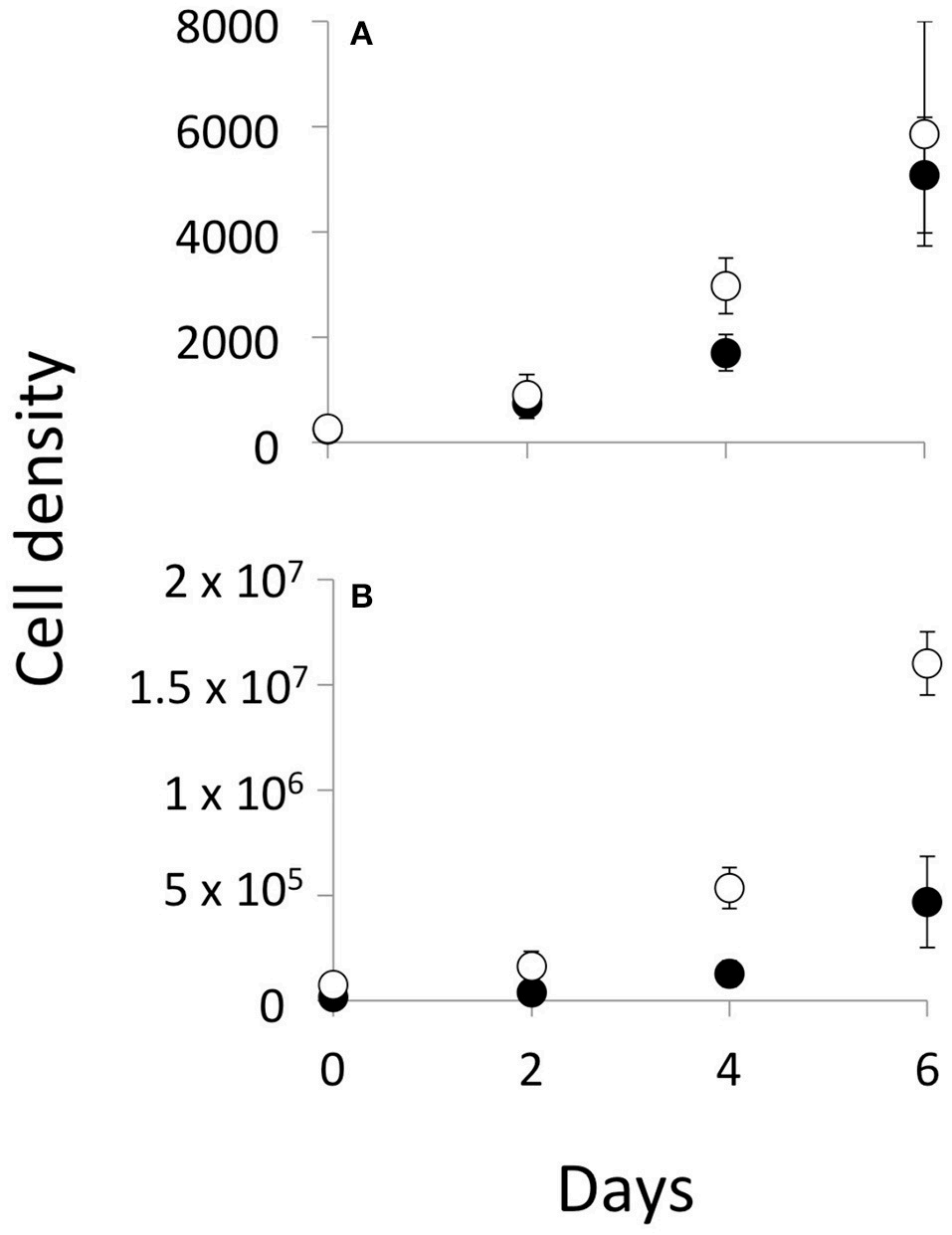
Temporal trend of mean cell density for *Cryptomonas* **(A)** and *Microcystis* **(B)** in the allelopathy experiment showing the mean cell density (cell.mL^−1^) measured in single culture controls (empty circles, *n* = 3) and co-cultured treatments (solid circles, *n* = 9). Error bars represent confidence interval at 95%.

**Figure 2:**
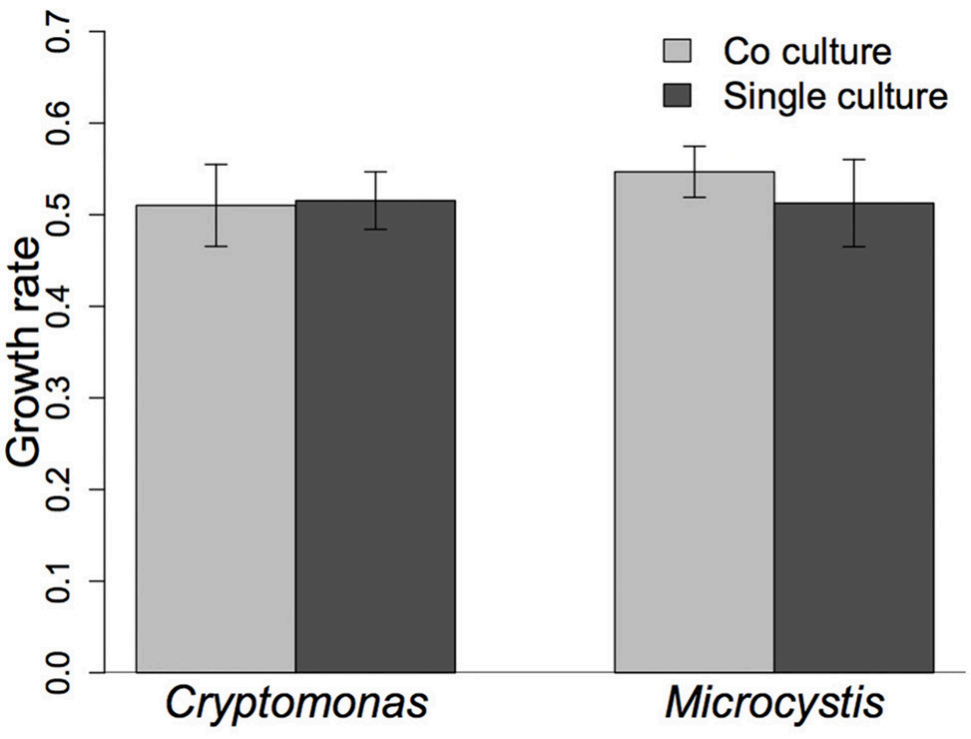
The mean net growth rate (cell.day^−1^) of *Cryptomonas* and *Microcystis* in the 6-day allelopathy experiment compared between the treatment (co-cultures, *n* = 9) and control (single culture, *n* = 3). Error bars indicate confidence intervals.

Both *Cryptomonas* (C) and *Microcystis* (M) grew exponentially during the experiment, regardless of the presence or abundance of a competitor (Figure 1). The initial C:M biomass ratio had no effect on the growth rate of either *Cryptomonas* (slope = −0.017 ± 0.03; *r*^2^ = 0.071; *p* = 0.245) or *Microcystis* (linear regression, slope = −0.002 ± 0.02; *r*^2^ = −0.129; *p* = 0.778, data not shown). We therefore grouped all C+M flasks into a single “co-culture” treatment (*n* = 9 for each phytoplankton). *Cryptomonas* growth rates were similar in single species controls and co-cultured treatment flasks (*t*-test; *t* = −0.18, *p* = 0.85), with a mean growth rate of 0.51 day^−1^ for both (± 0.03 and ± 0.05, *n* = 3 and 9, respectively) (Figure 2). Growth rates of *Microcystis* were also similar in single species cultures (control) and co-cultured treatments (*t*-test; *t* = 1.21, *p* = 0.29), with a mean growth rate of 0.51 day^−1^ (± 0.04, *n* = 3) in single cultures and 0.54 day^−1^ (± 0.03, *n* = 9) in co-cultures (Figure 2). Hence, growth rates of either phytoplankton were similar between controls and treatments and the presence of a competitor had no effect on growth.

### Facilitation

**Figure 3:**
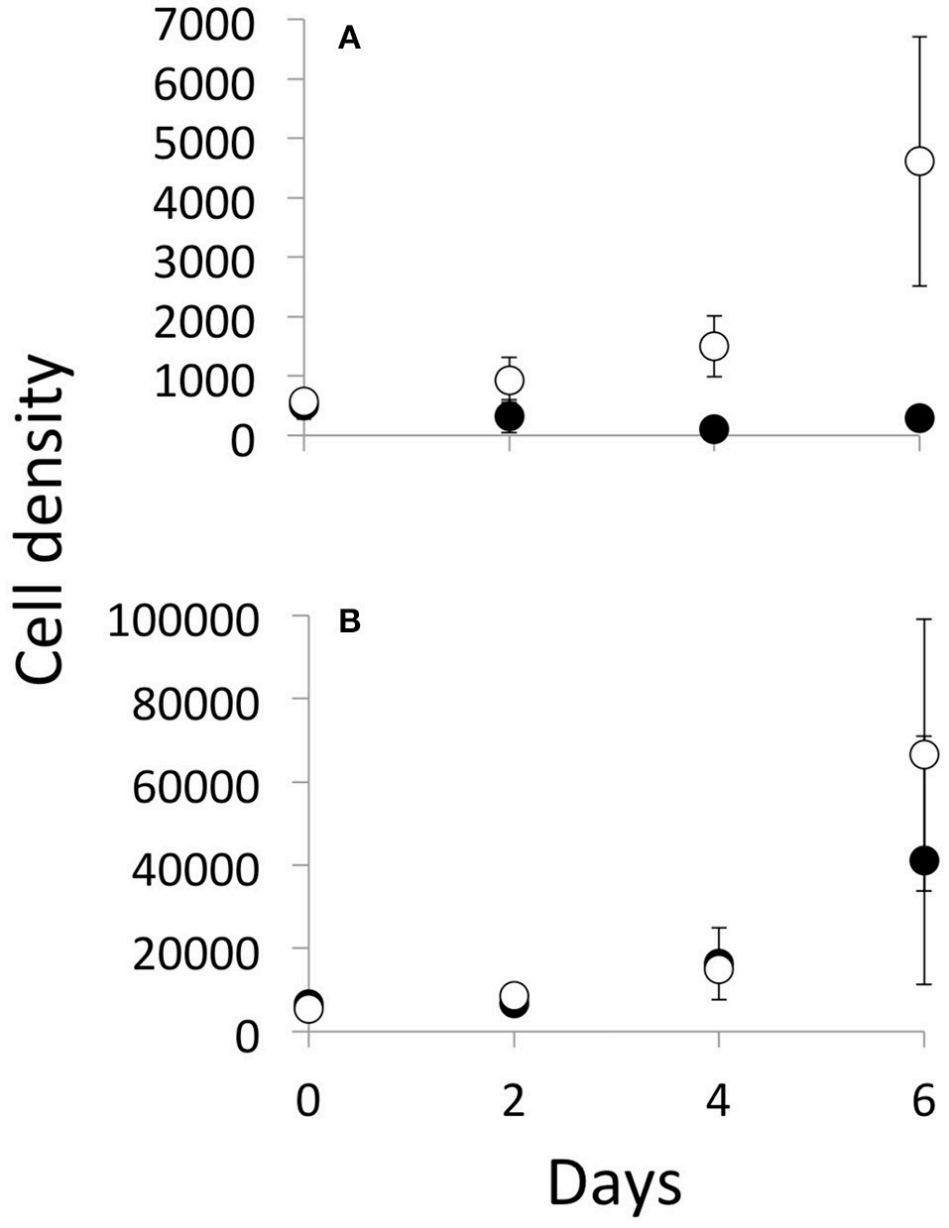
Temporal trend of mean cell density for *Cryptomonas* **(A)** and *Microcystis* **(B)** in the facilitation experiment showing the mean cell density (cell.mL^−1^) measured in no-grazer controls (empty circles, *n* = 4) and treatments with grazers (solid circles, *n* = 4). Error bars represent confidence interval at 95%.

**Figure 4:**
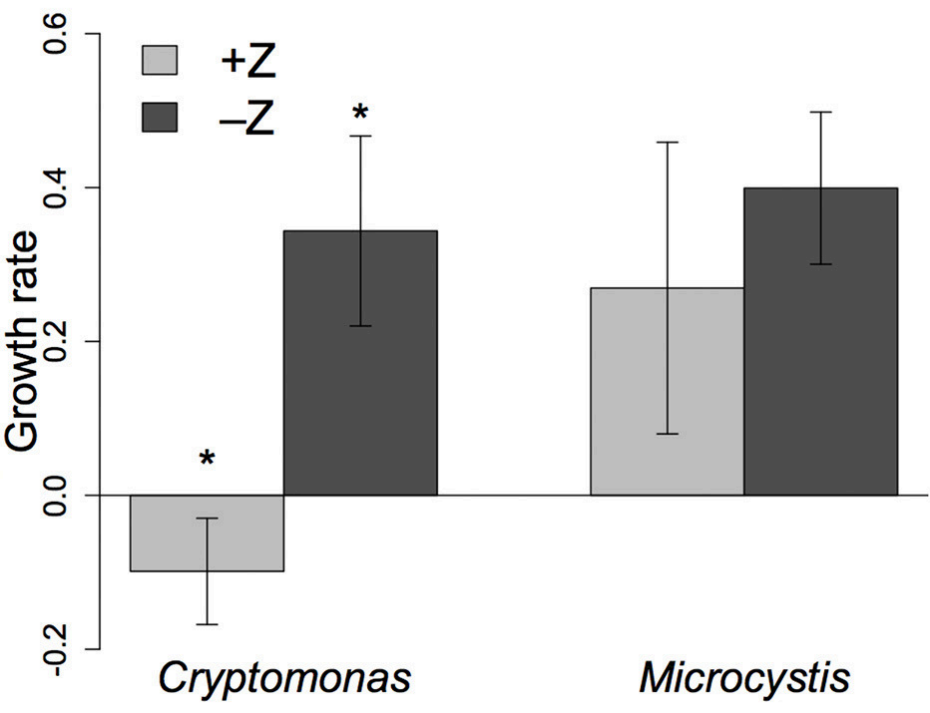
The mean net growth rate (cell.day^−1^) of each phytoplankton in 6-day facilitation experiment compared between treatments with copepods (+Z, *n* = 4) and no-grazer controls (–Z, *n* = 4). Error bars show confidence interval. ^*^Represents a significant difference (i.e., *p* < 0.05) between control and treatment.

Cell density of *Cryptomonas* increased exponentially in the no grazer control but decreased in the treatment with grazers (Figure 3A). Mean growth rate of *Cryptomonas* in the –Z controls was 0.34 day^−1^ (± 0.12, *n* = 4), compared to −0.09 day^−1^ (± 0.07, *n* = 4) in the +Z treatment (Figure 4). Hence, *Cryptomonas* growth rate was negatively affected by the presence of *N. iheringi* (*t*-test; *t* = −6.1304, *p* = 0.002). In contrast, cell density of *Microcystis* increased exponentially in both –Z controls and +Z treatments (Figure 3B). Moreover, while *N. iheringi* reduced *Microcystis* growth from a mean of 0.40 day^−1^ in –Z controls (± 0.10, *n* = 4) to mean of 0.27 day^−1^ (± 0.19, *n* = 4) in +Z treatments, this difference was not statistically significant (*t*-test; *t* = −1.19, *p* = 0.29, Figure 4). Thus, *Microcystis* continued to grow exponentially in the +Z treatments while *Cryptomonas* did not.

**Figure 5:**
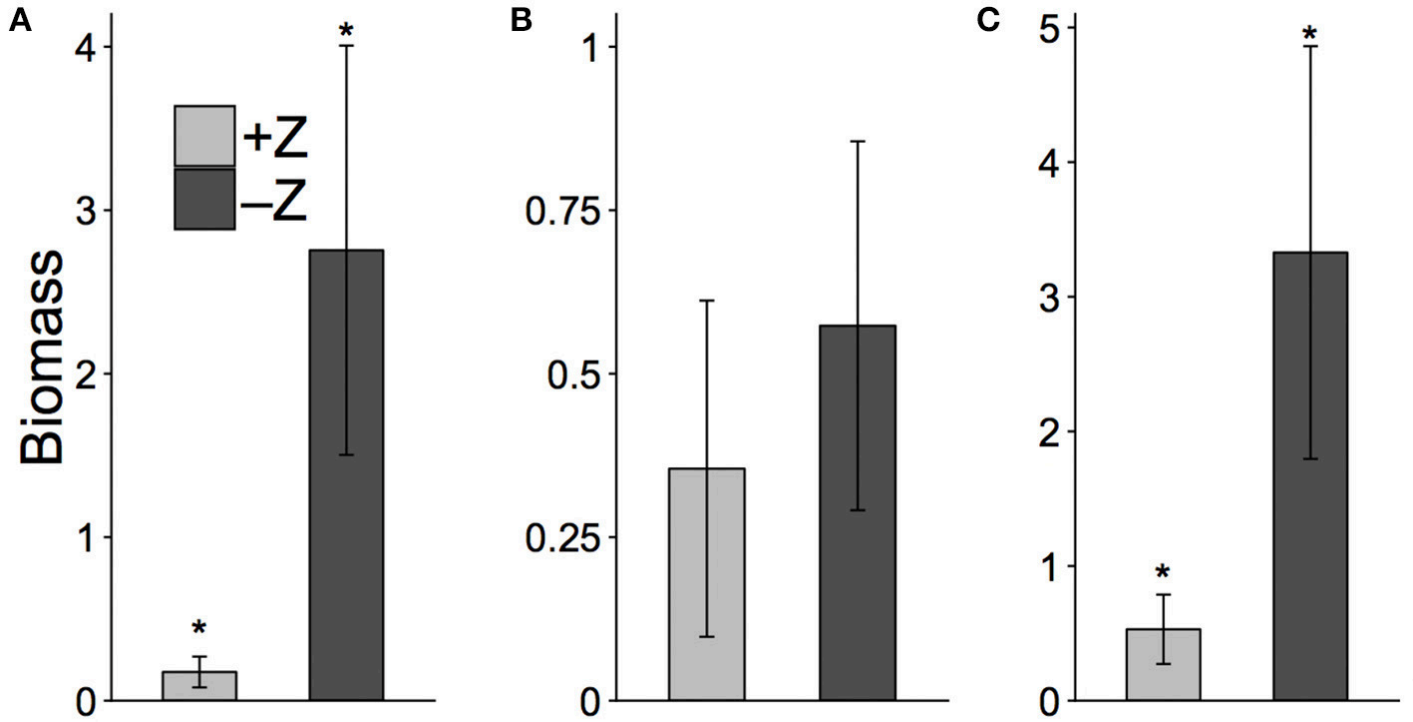
Mean biomass concentration (mgC.L^−1^) of *Cryptomonas* **(A)**, *Microcystis* **(B)**, and total phytoplankton **(C)** in the treatments with copepods (+Z) and no-grazer controls (–Z), at end of facilitation experiment (day 6). Error bars show 95% confidence intervals. ^*^Represents a significant difference (i.e., *p* < 0.05) between control and treatment.

The mean final biomass of *Cryptomonas* was reduced 15-fold in the presence of zooplankton when compared to –Z controls (Figure 5A, Kruskal-Wallis; χ^2^ = 4.50; df = 1; *p* = 0.03). In contrast, the mean final biomass of *Microcystis* was reduced by a factor of <2 in the presence of zooplankton, which was not significantly different than the no-grazer controls (Figure 5B, *t*-test, *t* = −1.449; df = 4.75; *p* = 0.20). Overall, grazing resulted in a significant, 6-fold decrease in the total phytoplankton biomass in treatments when compared to controls (Figure 5C, *t*-test, *t* = −3.53; df = 3.305; *p* = 0.033).

**Figure 6:**
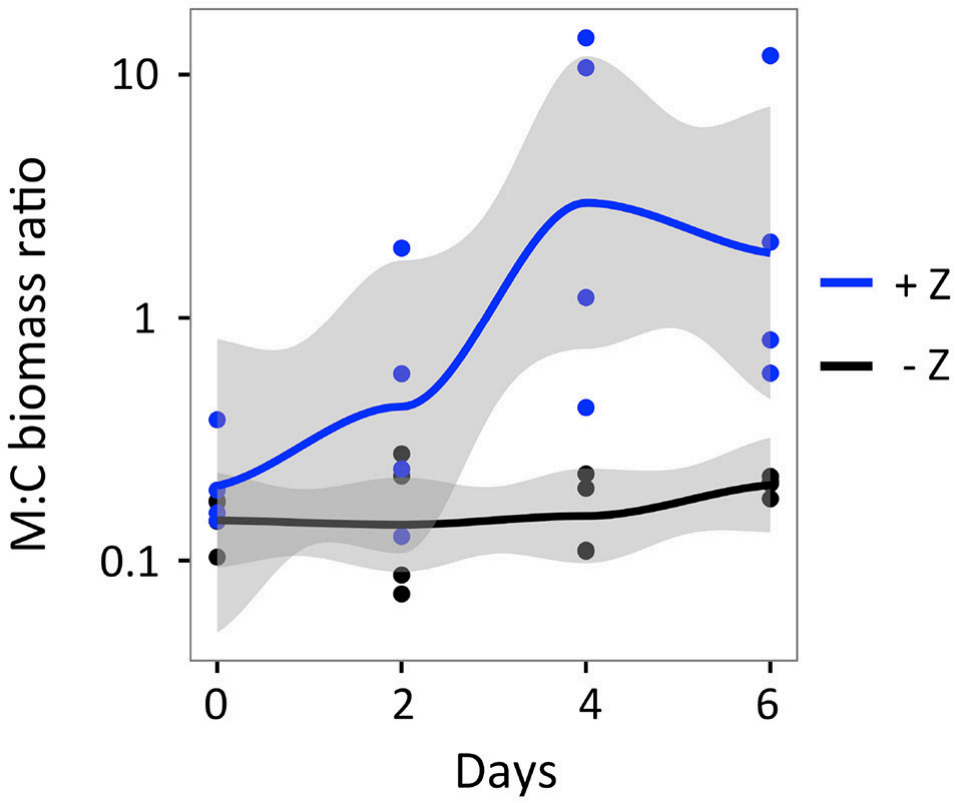
Change in the biomass ratio of *Microcystis* to *Cryptomonas* during the 6-day facilitation experiment compared between treatments with copepods (+Z) and no-grazer controls (–Z). Lines indicate the mean biomass ratio and the shaded area indicates the confidence interval (level = 95%).

The presence of copepods increased the relative abundance of *Microcystis* (i.e., M:C biomass ratio) when compared to the controls. While the M:C biomass ratio increased in both no grazer controls and treatments with grazers during the experiment, the increase was much stronger in the treatments (Figure 6). In the no-grazer controls, the mean growth of *Microcystis* (0.40 day^−1^) was similar yet higher than that of *Cryptomonas* (0.34 day^−1^) as detailed above. Thus, the mean change in the M:C biomass ratio increased in the controls by a factor of 1.4, from an initial mean of 0.15 (± 0.03, *n* = 4) to a final mean of 0.21 (± 0.02, *n* = 4) at day 6 (*t*-test, *t* = −2.88; df = 4.75; *p* = 0.038). In contrast, the mean net change in the M:C ratio increased in the treatments by a factor of about 13.3, from an initial mean of 0.21 (± 0.11, *n* = 4) to 3.85 (± 5.33, *n* = 4) at day 6 (Kruskal-Wallis; χ^2^ = 5.33; df = 1; *p* = 0.021). Hence, despite high variance in the net change of the treatment M:C ratios, *N. iheringi* significantly increased the M:C ratio in each replicate flask compared to controls at days 4 and 6 (Figure 6, Table 1). Moreover, *N. iheringi* reversed the initial dominance from *Cryptomonas* to *Microcystis* in two out of four of the treatment flasks by the end of the experiment. Overall, grazing by *N. iheringi* increased the M:C ratio by a factor of 9.4 when compared to controls (Figure 6).

**Table 1:** Statistical summary comparing differences in the *Microcystis* to *Cryptomonas* biomass ratio (M:C) among controls (no-grazers) and treatments (with-grazers) for each experimental day in the facilitation experiment as evaluated by Kruskal-Wallis tests.

**Day**	**χ^2^**	***n***	**df**	***p***
2	2.08	8	1	0.149
4	5.33	8	1	0.021
6	5.33	8	1	0.021

**Figure 7:**
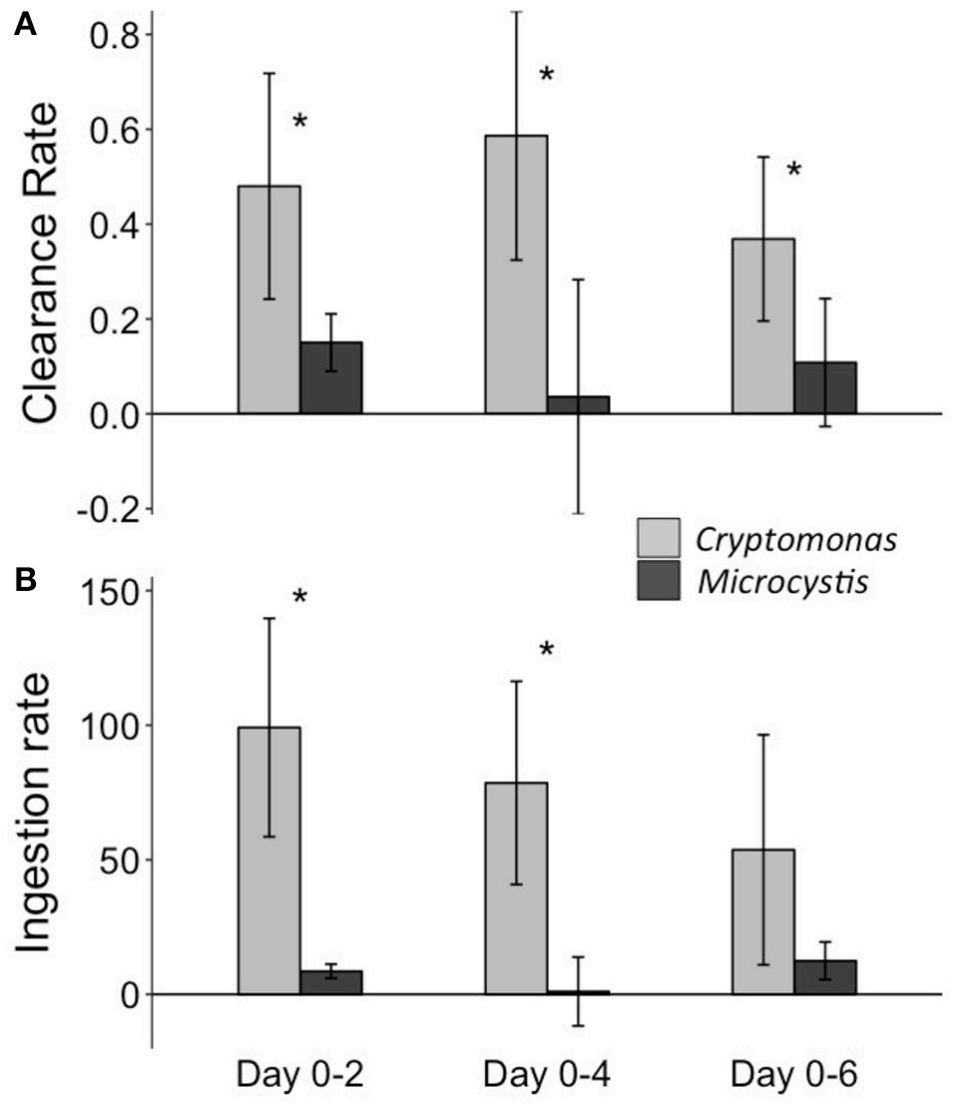
Mean prey specific rates of **(A)** clearance (mL.copepod^−1^.hour^−1^) and **(B)** ingestion (ng.copepod^−1^.hour^−1^) of *N. iheringi* on *Cryptomonas* and *Microcystis*, measured across different time periods (i.e., 0–2, 0–4, 0–6 days) during the facilitation experiment. Error bars show the 95% confidence intervals. ^*^Represents a significant difference (i.e., *p* < 0.05) between grazing rates on each prey type for a given time period.

The mean clearance rate of *N. iheringi* on *Cryptomonas* (CR_C_) was higher than on *Microcystis* (CR_M_) by a factor of three or more throughout the experiment (Figure 7A). When measured over the entire 6-day period, mean CR_C_ was 0.36 ml.copepod^−1^ h^−1^ (± 0.11, *n* = 4), while mean CR_M_ was 0.11 copepod mL^−1^ h^−1^ (± 0.08, *n* = 4) and this difference was significant (Appendix Table 2). Mean prey specific ingestion rate on *Cryptomonas* (IR_C_) was significantly higher than on *Microcystis* (IR_M_) by a factor of at least 10 when measured during the period of 0–2 and 0–4 days, but no significant difference was found when measured over the entire 6-day period (Figure 7B, Appendix Table 2). Mean prey specific clearance rates were relatively stable and comparable across the different time periods over which they were measured [CR_C_: *F*_(2, 9)_ = 2.31, *p* = 0.15 and CR_M_: *F*_(2, 9)_ = 1.23, *p* = 0.34]. Mean prey specific ingestion rates were also relatively stable across different time periods [IR_C_: *F*_(2, 9)_ = 1.21, *p* = 0.34 and IR_M_: χ^2^ = 2.80, df = 2, *p* = 0.24 (Kruskal-Wallis)], although mean IR_C_, showed a slight decrease with time.

Copepod mortality was less than 4% and 7 out of 200 individuals were found dead. Copepods also produced eggs and possibly nauplii, which were not counted. *Microcystis* cells showed no change in morphology and were found as single or bi-cells. Phytoplankton growth rates in co-cultures without grazers (i.e., allelopathy treatment and facilitation control) were either constant or slightly increased over time when compared during the sub-sampling intervals of 0–2; 2–4; and 4–6 days (Figure 1, Appendix Table 3, Appendix Figure 1).

## Discussion

We evaluated the interaction of a common bloom-forming cyanobacterium with a eukaryotic phytoplankton competitor in the presence and absence of a copepod grazer to test whether zooplankton could facilitate cyanobacterial dominance. Adding the copepod *N. iheringi* to the competing phytoplankton increased the relative abundance of *Microcystis* co-cultured with *Cryptomonas* compared to no-grazer controls. Strong grazing pressure reduced the abundance of *Cryptomonas*, which resulted in negative growth rates for this phytoplankton. In contrast, neither the abundance nor the growth of *Microcystis* was affected significantly by *N. iheringi*. Overall, these results indicate that *N. iheringi*, a copepod commonly associated to cyanobacterial blooms in nature, facilitated the dominance of *Microcystis* by selectively consuming *Cryptomonas*. Hence, our results provide unique experimental evidence that zooplankton co-occurring with blooms may promote cyanobacterial dominance by selective grazing on their eukaryotic phytoplankton competitors. We believe these are novel results with implications for the role of selective grazers on cyanobacterial bloom dynamics and plankton ecology in general, especially given the future scenarios of higher temperatures and increased eutrophication (Paerl and Huisman, 2009).

The results emphasize the role of biotic drivers for facilitating the dominance of cyanobacteria and possibly other taxa of inedible phytoplankton. Top-down regulation of the phytoplankton community composition by zooplankton, especially by large generalist grazers like *Daphnia*, has been recognized for decades (Bergquist et al., 1985; Sterner, 1989). Increased grazer pressure, especially by large cladocerans, can shift the phytoplankton community to inedible chlorophytes or cyanobacteria with a colonial gelatinous morphology (Shapiro and Wright, 1984; Carpenter et al., 1987). Considerable research has also focused on the ability of zooplankton to control phytoplankton biomass and especially cyanobacterial blooms (e.g., Jeppesen et al., 2007). Yet little is known about the potential for zooplankton to positively affect cyanobacteria. Indeed, to our knowledge there is no previous experimental evidence besides short-term grazing experiments (Jiang et al., 2013; Ger et al., 2016b), though modeling studies (Mitra and Flynn, 2006) and associations from mesocosms and monitoring data (Hong et al., 2015; Rangel et al., 2016) have suggested positive interactions among selective grazing zooplankton and cyanobacteria. Hence, by scaling up from short-term grazing assays to using artificial foodwebs, our results provide further evidence for a plausible mechanism explaining the co-existence of copepods and cyanobacterial blooms. The top-down effect of smaller, selectively grazing zooplankton on the structure and function of phytoplankton community, however, remains poorly understood (Ger et al., 2016c), which limits predictions for the ecology of a warmer and more eutrophic world where these zooplankters are expected to dominate.

Selective grazing on *Cryptomonas* and avoidance of *Microcystis* by the copepod *N. iheringi* was likely due to a combination of toxicity and poor nutrition. Such behavior relies on cues to detect the size, shape, chemical composition, and toxicity of potential prey to maximize nutritional gain and minimize ingestion of harmful prey (DeMott and Moxter, 1991; Kleppel, 1993; Heuschele and Selander, 2014). The nutritional quality of phytoplankton for zooplankton grazers is largely based on sterols and fatty acids (Muller-Navarra et al., 2000; von Elert et al., 2003; Ravet et al., 2010). Cyanobacteria, including *Microcystis*, are low in nutrition while *Cryptomonas* is nutritious with a high concentration of highly unsaturated fatty-acids (Brett and Müller-Navarra, 1997). Copepods can avoid ingesting cyanobacteria by sensing chemical cues (e.g., toxins), while maintaining uninhibited ingestion on alternative prey (DeMott, 1986). Indeed, phytoplankton anti-grazer defenses also regulate zooplankton grazing and copepod prey selection (Pohnert et al., 2007). Both toxins and colonial morphology have been shown to defend phytoplankton against zooplankton grazers (Fulton and Paerl, 1988; Selander et al., 2006). In this study, the toxicity of the *Microcystis* strain likely explains copepod avoidance as the single or bi-cellular nature of the cultured *Microcystis* rules out colonial morphology as a defensive trait. Indeed, previous studies have suggested that microcystins may act as anti-grazer defenses for copepods including *N. iheringi* (Ger et al., 2016a,b). Moreover, although copepods are known to select for larger sized particles (DeMott et al., 1991), *N. iheringi* ingestion of a smaller (3 μm) non-microcystin producing *Microcystis* strain was 3-fold higher than a larger (5 μm) microcystin-producing strain (Ger et al., 2016b). Thus, the reason why *Microcystis* was avoided in this study was likely prey chemistry (i.e., toxicity), not size. In contrast with our study, however, *Microcystis* and other bloom-forming cyanobacteria exist as larger colonies or filaments in nature. Future work on grazer facilitation of cyanobacteria would therefore benefit from accounting for morphological defenses in similar incubations.

Rates of *Microcystis* clearance by *N. iheringi* in our study were similar to previous short-term (i.e., 2 h) grazing assays with the same phytoplankton prey species (Ger et al., 2016b). Clearance rates were also similar to other short-term experiments with copepods and bloom-forming cyanobacteria measured using a variety of methods, zooplankton density, and volumes ranging from 2.5 mL to 0.5 L (DeMott and Moxter, 1991; Ger et al., 2011; Hong et al., 2013). While these results indicate that grazing rates obtained from short-term assays may also reflect longer-term dynamics, it is likely that multiple factors affect longer term grazing in this current experimental setup. The relative concentration of prey to grazers should be a key factor regulating prey switching (Kiørboe et al., 1996) and extreme depletion of one prey (e.g., *Cryptomonas*) may result in increased grazing on *Microcystis*. Yet, prey specific clearance or ingestion rates measured over 2, 4, and 6 days were similar, suggesting that *N. iheringi* grazing on each prey type was relatively constant during our experiment. Hence, although the abundance of *Cryptomonas* was declining and that of *Microcystis* was increasing, *N. iheringi* maintained higher clearance or ingestion rates for the former and we found no evidence for prey switching during the 6-day incubation. Longer-term incubations, however, would help understand whether copepods completely deplete edible phytoplankton and how rapid and persistent prey switching is given the shifting dominance of poor quality prey such as toxic cyanobacteria.

The fact that copepods facilitate the dominance of cyanobacteria by ingesting edible phytoplankton raises the question how these grazers maintain high fitness during blooms when edible phytoplankton is scarce. If the nutritious edible prey were completely depleted, zooplankton fitness would be expected to decline as a result of food limitation or toxicity from switching to cyanobacterial prey (Sommer et al., 1986). Indeed, the role of nutritious prey for controlling copepod fitness was shown previously and although *N. iheringi* tolerated and produced eggs in diets dominated by toxic *Microcystis*, fitness was a function of nutritious food (Ger et al., 2016b).

The question of how copepods such as *N. iheringi* reach high biomass during blooms of toxic cyanobacteria in nature (e.g., Rangel et al., 2016), however, was outside the scope of this study. One plausible explanation is that copepods are omnivores and efficiently select for optimal prey including heterotrophic protists and fungal parasites, which are known to graze on and upgrade the food quality of toxic cyanobacteria (Engström-Öst et al., 2002; Park et al., 2003; Bec et al., 2006; Agha et al., 2016). Hence, even in a complete absence of edible and nutritious phytoplankton during long-duration cyanobacteria blooms, microzooplankton grazers can be expected to provide a sufficient and nutritious food source to explain high copepod fitness. Given that heterotrophic protists are expected to graze on cyanobacteria and be grazed by crustacean zooplankton (Canter et al., 1990; Engström-Öst et al., 2002; Bec et al., 2006; Combes et al., 2013), their intermediary role in fueling zooplankton production in bloom dominated waters merits more attention.

The current results emphasize how selective grazers may facilitate cyanobacterial dominance, at least in the absence of resource limitation. Indeed, the current study was designed to test the top-down effects of selective grazers under no resource limitation. We found no evidence for allelopathy, nutrient or light limitation as the growth rate of either *Microcystis* or *Cryptomonas* was unaffected by the presence or proportion of their competitor during the experiment and growth rates were constant or increasing for both phytoplankton in the absence of grazers. The difference in the growth rates of co-cultured phytoplankton in the allelopathy experiment and the no grazer control of the facilitation experiment were likely due to differences in the experimental setup (e.g., 250 mL vs. 1 L flasks, cotton wool vs. silicone stoppers with bubbling, see methods for details). Despite such differences, there was no evidence for allelopathy or another type of resource competition that could explain the grazer effect found in the current setup. Yet, top-down grazer effects on cyanobacterial dominance in nutrient or light limited waters may be different than what the current results suggest. Allelopathy is commonly observed among cyanobacteria and eukaryotic phytoplankton, including *Microcystis* and *Cryptomonas* (B-Béres et al., 2012; Mello et al., 2012; Rzymski et al., 2014; Bittercourt-Oliveira et al., 2015; Ma et al., 2015), and nutrient limitation can induce the production of allelopathic compounds by cyanobacteria and reducing the fitness of competing phytoplankton (Sukenik et al., 2002; Xu et al., 2017). Resource limitation can also result in indirect grazer effects on phytoplankton communities such as nutrient regeneration by zooplankton excretion (Elser et al., 1988). Zooplankton excretion may alter the concentration and ratio of nutrients available to phytoplankton due to phylum or species specific differences in the elemental composition (Sterner et al., 1992). Consequently, future work would benefit from considering both bottom-up and top-down effects simultaneously to evaluate the role of selective grazers on cyanobacterial abundance across a variety of abiotic conditions.

In addition to resource competition, future work would also benefit from accounting for indirect grazer effects on phytoplankton competition by inducing chemical or morphological changes in their prey. Chemical grazer cues may result in increased toxin formation or morphological defenses in phytoplankton (Jang et al., 2007; Yang et al., 2008). Since *Microcystis* morphology did not change in the presence of grazers in this experiment, it is not a likely factor explaining the results. Yet it is possible that toxin production in *Microcystis* increased due to grazing (Jang et al., 2007). Although the role of grazer induced chemical defenses on the ecology of phytoplankton is poorly understood (Pohnert et al., 2007), increased production of microcystins reduced copepod grazing pressure on *Microcystis* in a recent study (Ger et al., 2016a). Thus, future studies would benefit from accounting for chemical (i.e., stoichiometry, cellular and dissolved toxins) and morphological changes when studying the effect of zooplankton on phytoplankton competition.

Our results emphasize the potential for top-down regulation of phytoplankton communities by selective grazers in warmer and more eutrophic waters. Given that the bulk of freshwater zooplankton work is based on large generalist grazers (i.e., *Daphnia*), which become more rare in warmer and more eutrophic water bodies (Elser and Goldman, 1991; Fernando, 1994; Auer et al., 2004; Jeppesen et al., 2011), the role of selective grazers on phytoplankton dynamics is still mostly unexplored. Thus, important questions remain before further predictions can be made regarding the ecological relevance of our results for a warmer and more eutrophic future world. For example, there are several distinct functional traits represented within selectively grazing zooplankton (e.g., raptorial feeders, feeding current, cruise feeding) (Litchman et al., 2013). Each group of selectively feeding zooplankton is further differentiated in terms of their nutritional requirements and tolerance to ingested cyanotoxins (Litchman et al., 2013; Ger et al., 2016c). Moreover, variability in cyanobacterial traits, including toxin production and morphology, is equally large (Kruk et al., 2010; O'Neil et al., 2012), which further limits predictions based on the results of this study. Hence, while the current results suggest a strong positive effect of selective grazing zooplankton on the dominance of *Microcystis*, we urge future work to account for different functional groups within zooplankton and cyanobacteria—especially those that co-exist in nature—when evaluating the top-down regulation of cyanobacterial blooms in a more eutrophic world.

In conclusion, our results suggest that selective avoidance of cyanobacteria by zooplankton grazers is likely an important mechanism regulating the dynamics and dominance of cyanobacterial blooms and phytoplankton community structure in general, at least in nutrient rich waters where bloom-forming cyanobacteria typically occur. Since smaller and more selective grazing zooplankton dominate in eutrophic waters, the potential for grazer facilitation of blooms may operate as a positive feedback that stabilizes blooms. Bloom facilitation by grazers may be a major factor explaining the association of high zooplankton biomass during long duration cyanobacterial blooms, especially in warmer eutrophic waters. However, as detailed above, the large variability in the functional traits of both grazers and cyanobacteria, as well as the intermediary role of heterotrophic protists and parasites as both grazers of cyanobacteria and prey to larger crustacean zooplankton highlight the need for considering more species interactions. Ultimately, while selective grazers may facilitate blooms, what sustains their elevated biomass during blooms of toxic phytoplankton will determine the structure and function of bloom-dominated waters (Ger et al., 2014).

## Author contributions

All authors designed the experiment and analyzed the data. All authors contributed to writing the experiment. EL and KG performed the experiment.

### Conflict of interest statement

The authors declare that the research was conducted in the absence of any commercial or financial relationships that could be construed as a potential conflict of interest.
