# Supplementary material for: Selective Grazing by a Tropical Copepod (Notodiaptomus iheringi) Facilitates Microcystis Dominance
Source: Front Microbiol. 2018 Feb 23;9:301. doi: 10.3389/fmicb.2018.00301 (PMC5829094; doi:10.3389/fmicb.2018.00301)

Appendix Table 1:The initial concentration of each replicate shown together with the mean and standard deviation in each control or treatment for both phytoplankton species in carbon equivalent biomass (mgC.L^-1^) in the allelopathy (**A**) and facilitation (**B**) experiments.

| Diet  Composition | *Cryptomonas* | | *Microcystis* | | Total | |
| --- | --- | --- | --- | --- | --- | --- |
|  | value | mean±SD | value | mean±SD | value | mean±SD |
| (A) *Allelopathy* |  |  |  |  |  |  |
| Control (single-culture) |  |  |  |  |  |  |
| *Cryptomonas* only | 0.172 | 0.159±0.02 | - | - | 0.172 | 0.159±0.02 |
| *Cryptomonas* only | 0.135 |  | - |  | 0.135 |  |
| *Cryptomonas* only | 0.169 |  | - |  | 0.169 |  |
| *Microcystis* only | - | - | 0.724 | 0.646±0.13 | 0.724 | 0.646±0.13 |
| *Microcystis* only | - |  | 0.725 |  | 0.725 |  |
| *Microcystis* only | - |  | 0.488 |  | 0.488 |  |
| Treatment (co-culture) |  |  |  |  |  |  |
| 0.21C:1M | 0.076 | 0.149±0.08 | 0.355 | 0.157±0.12 | 0.431 | 0.307±0.11 |
| 0.22C:1M | 0.073 |  | 0.323 |  | 0.396 |  |
| 0.63C:1M | 0.169 |  | 0.264 |  | 0.433 |  |
| 1.12C:1M | 0.124 |  | 0.110 |  | 0.234 |  |
| 1.37C:1M | 0.136 |  | 0.098 |  | 0.234 |  |
| 1.81C:1M | 0.191 |  | 0.105 |  | 0.296 |  |
| 2.40C:1M | 0.114 |  | 0.047 |  | 0.161 |  |
| 2.67C:1M | 0.129 |  | 0.048 |  | 0.178 |  |
| 5.55C:1M | 0.338 |  | 0.061 |  | 0.398 |  |
|  |  |  |  |  |  |  |
| (B) *Facilitation* |  |  |  |  |  |  |
| Control (no-grazer) |  |  |  |  |  |  |
| 5.68C:1M | 0.239 | 0.342±1.22 | 0.042 | 0.048±0.01 | 0.281 | 0.390±0.12 |
| 5.75C:1M | 0.248 |  | 0.043 |  | 0.291 |  |
| 9.67C:1M | 0.497 |  | 0.051 |  | 0.548 |  |
| 6.89C:1M | 0.383 |  | 0.055 |  | 0.438 |  |
| Treatment (w/ grazer) |  |  |  |  |  |  |
| 2.64C:1M | 0.109 | 0.301±0.14 | 0.041 | 0.055±0.01 | 0.150 | 0.357±0.15 |
| 6.37C:1M | 0.278 |  | 0.043 |  | 0.321 |  |
| 5.13C:1M | 0.387 |  | 0.075 |  | 0.462 |  |
| 6.84C:1M | 0.432 |  | 0.063 |  | 0.496 |  |

Appendix Table 2: Results of t-tests comparing the log-transformed differences in the clearance or ingestion rate of *Cryptomonas* and *Microcystis* by the copepod *N. iheringi* measured across each time period.

| Period  (days) | Clearance rate | | |  | Ingestion rate | | |
| --- | --- | --- | --- | --- | --- | --- | --- |
|  | *t* | df | *p* |  | *t* | df | *p* |
| 0-2 | 5.24 | 5.78 | 0.002 |  | 4.36 | 3.02 | 0.022 |
| 0-4 | 4.84 | 5.97 | 0.002 |  | 3.81 | 3.67 | 0.022 |
| 0-6 | 3.78 | 5.66 | 0.010 |  | 1.87 | 3.16 | 0.150 |

Appendix Table 3: The mean intercept and slope of biomass increase over different time periods (via log-transformed general linear models) of each co-cultured phytoplankton in the allelopathy and facilitation experiment in the absence of grazers.

The slope indicates the growth rate across the total length of experiment of 6 days (0-6), compared to segmented 2-day periods when sub-sampling occurred (i.e., 0-2; 2-4; 4-6). CI indicates the 95% confidence interval for each mean value.

|  | Intercept±CI | Slope±CI |
| --- | --- | --- |
| Allelopathy |  |  |
| *Cryptomonas* |  |  |
| 0-6 | -2.002±1.039 | 0.510±0.134 |
| 0-2 | -2.002±0.347 | 0.533±0.246 |
| 2-4 | -1.846±0.671 | 0.455±0.212 |
| 4-6 | -2.195±0.846 | 0.542±0.166 |
| *Microcystis* |  |  |
| 0-6 | -2.127±0.545 | 0.547±0.128 |
| 0-2 | -2.127±0.576 | 0.375±0.407 |
| 2-4 | -2.589±1.285 | 0.606±0.406 |
| 4-6 | -2.801±1.961 | 0.659±0.384 |
| Facilitation |  |  |
| *Cryptomonas* |  |  |
| 0-6 | -0.121±0.480 | 0.346±0.113 |
| 0-2 | -1.121±0.497 | 0.232±0.352 |
| 2-4 | -1.152±1.146 | 0.247±0.133 |
| 4-6 | -2.366±1.786 | 0.551±0.350 |
| *Microcystis* |  |  |
| 0-6 | -3.043±0.437 | 0.399±0.103 |
| 0-2 | -3.043±0.240 | 0.250±0.170 |
| 2-4 | -3.044±0.493 | 0.251±0.156 |
| 4-6 | -4.830±1.537 | 0.697±0.301 |

Appendix Figure 1: The mean biomass (µgC.mL^-1^) of each co-cultured phytoplankton in the allelopathy (a) and the facilitation (b) experiment in the absence of grazers during the 6-day incubation period. Empty and solid circles show mean biomass of *Cryptomonas* and *Microcystis*, respectively, while the error bars indicate the 95% confidence interval. .


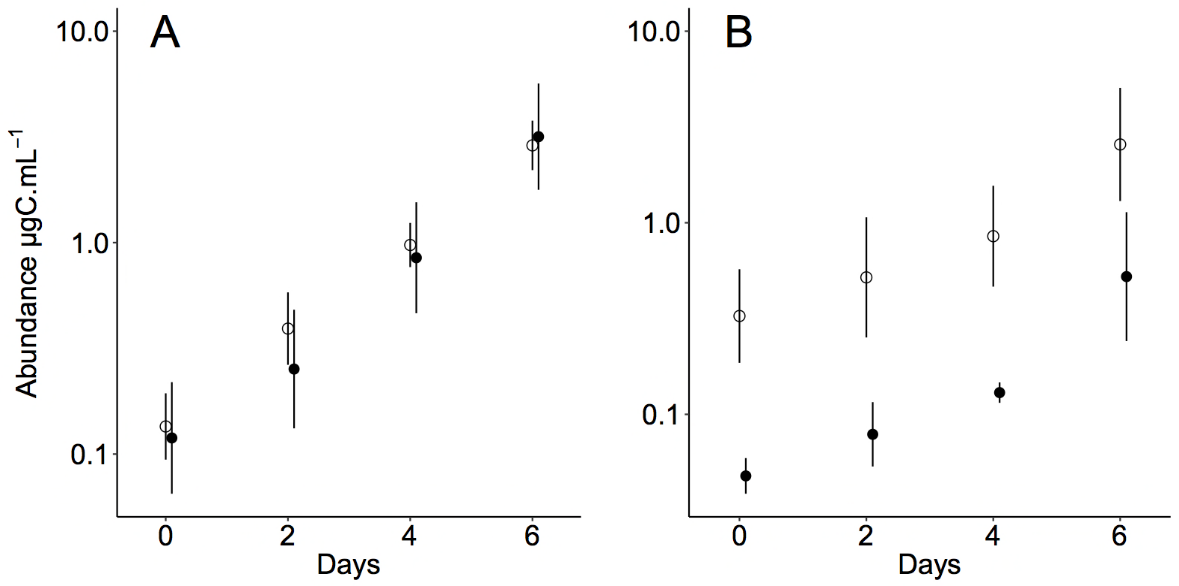

Supplement: Supplementary file 1 [file SupplementaryMaterial.docx]
